# Antioxidant Activity and Cytotoxicity against Cancer Cell Lines of the Extracts from Novel *Xylaria* Species Associated with Termite Nests and LC-MS Analysis

**DOI:** 10.3390/antiox10101557

**Published:** 2021-09-29

**Authors:** Niwana Wangsawat, Lutfun Nahar, Satyajit D. Sarker, Cherdchai Phosri, Andrew R. Evans, Anthony J. S. Whalley, Kiattawee Choowongkomon, Nuttika Suwannasai

**Affiliations:** 1 Department of Biology, Faculty of Science, Srinakharinwirot University, Bangkok 10110, Thailand; niwana.wangsawat@g.swu.ac.th; 2 Laboratory of Growth Regulators, Institute of Experimental Botany ASCR & Palacký University, Šlechtitelů 27, 78371 Olomouc, Czech Republic; drnahar@live.co.uk; 3 Centre for Natural Products Discovery (CNPD), School of Pharmacy and Bimolecular Sciences, Liverpool John Moores University, James Parsons Building, Byrom Street, Liverpool L3 3AF, UK; S.Sarker@ljmu.ac.uk (S.D.S.); A.R.Evans@ljmu.ac.uk (A.R.E.); A.J.Whalley@ljmu.ac.uk (A.J.S.W.); 4 Department of Biology, Faculty of Science, Nakhon Phanom University, Nakhon Phanom 48000, Thailand; cherd.phosri@npu.ac.th; 5 Department of Biochemistry, Faculty of Science, Kasetsart University, Bangkok 10900, Thailand; kiattawee.c@ku.ac.th; 6 Center for Advanced Studies in Nanotechnology for Chemical, Food and Agricultural Industries, KU Institute for Advanced Studies, Kasetsart University, Bangkok 10900, Thailand; 7 Department of Microbiology, Faculty of Science, Srinakharinwirot University, Bangkok 10110, Thailand

**Keywords:** antioxidant, cytotoxicity, LC-MS fingerprint, *Xylaria*, termite nest

## Abstract

*Xylaria* species associated with termite nests or soil have been considered rare species in nature and the few which have been reported upon have been found to act as a rich source of bioactive metabolites. This study evaluated 10 ethyl acetate extracts of five new *Xylaria* species associated with termite nests or soil for their antioxidant activity, and cytotoxicity against different cancer and normal cell lines. DPPH and ABTS radical scavenging activities of the extracts demonstrated strong capacity with low IC_50_ values. The highest observed activities belonged to *X. vinacea* SWUF18-2.3 having IC_50_ values of 0.194 ± 0.031 mg/mL for DPPH assay and 0.020 ± 0.004 mg/mL for ABTS assay. Total phenolic content ranged from 0.826 ± 0.123 to 3.629 ± 0.381 g GAE/g crude extract which correlated with antioxidant activities. The high total phenolic content could contribute to the high antioxidant activities. Cytotoxicity was recorded against A549, HepG2, HeLa and PNT2 and resulted in broad spectrum to specific activity depending on the cell lines. The highest activities were observed with *X. subintraflava* SWUF16-11.1 which resulted in 11.15 ± 0.32 to 13.17 ± 2.37% cell viability at a concentration of 100 µg/mL. Moreover, LC-MS fingerprints indicated over 61 peaks from all isolates. There were 18 identified and 43 unidentified compounds compared to mass databases. The identified compounds were from various groups of diterpenoids, diterpenes, cytochalasin, flavones, flavonoids, polyphenols, steroids and derivatives, triterpenoids and tropones. These results indicate that *Xylaria* spp. has abundant secondary metabolites that could be further explored for their therapeutic properties.

## 1. Introduction

Reactive oxygen species (ROS) is the most important class of free radicals normally generated in living systems and it plays essential roles resulting in positive or negative effects in cells. Low or suitable concentrations of ROS can stimulate signaling pathways in cells to respond to various physiological functions such as immune function (i.e., defense against pathogenic microorganisms), mitogenic response and redox regulation [1,2]. The imbalance of ROS production is extremely harmful to organisms at high concentrations. When the level of ROS exceeds the defence mechanisms, generally known as oxidative stress, it can affect many cellular functions by damaging lipids, proteins and nucleic acids, and it appears to be a major contributor to degenerative diseases such as cancer, diabetes mellitus, neurological disorders, cardiovascular diseases, cataracts, immune system decline, renal failure and mutagenesis [3,4,5]. Neutralizing free radicals by antioxidant substances is therefore important for cell protection and therapeutics uses [6]. Antioxidants are chemicals which inhibit the oxidation reaction of free radicals by exchanging one of their own electrons with the free radical molecules to stabilize them.

Cancer is the second leading cause of death worldwide, accounting for an estimated 9.6 million deaths, behind cardiovascular disease. Lung, colorectal, liver, breast and cervical cancer have been the most common types of cancer in humans during the past decade (WHO 2018) [7]. Cancer treatment involves a number of approaches which include surgical intervention, chemotherapy and radiation therapy or often a combination of these treatments [8]. There has been considerable success using natural compounds as part of the current chemotherapeutic arsenal. This has stimulated the exploration for novel natural compounds from relatively vast and untapped resources found in nature. Various natural resources including plants and fungi have been found to produce many biologically active substances including those with potent antioxidants and anticancer properties. Fungi are a diverse and valuable source of enormous chemical potential. They are of major interest because only a small percentage of them have been investigated for their role in producing novel bioactive compounds and hence offer huge potential.

The *Xylaria* is a large genus in the fungal family Xylariaceae, which is known to be a rich source of secondary metabolites—e.g., phenolic acids, flavonoids, alkaloids, steroids, polyketides, amino amidine, cytochalasins, ergot alkaloids, ergosterol, indole alkaloids and etc. [9,10,11,12]. Over the last few decades, several new bioactive compounds have been regularly discovered from *Xylaria* species and found to exhibit effective antimicrobial, antifungal, anti-inflammatory, antioxidant and anticancer activities [13,14]. However, most *Xylaria* species investigated were obtained as endophytes or saprophytes on fallen woods. There are only a few reports published on bioactive compounds from *Xylaria* species associated with termite nests or grown on soil because they were rarely found and difficult to culture [15,16,17,18,19]. The termite nest-derived *X. nigripes*, also known as “Wu Ling Shen”, is one of the most famous fungi in use as a traditional Chinese medicine for treating insomnia, trauma, cognitive deficits, depression, and anemia [20]. The ethyl acetate extracts of the fermented broths of *X. nigripes* have been found to exhibit antioxidant and anti-inflammatory activities [21], and to enhance the immunity and hematopoiesis [22]. Moreover, the study of the medicinal fungus *X. fimbriata* obtained from termite nests revealed high anti-inflammatory activity with seven compounds of isoprenyl phenolic ethers from the ethyl acetate extracts [23]. Recently, we published several new species of *Xylaria* associated with termite nests from Thailand together with their pure culture collections [19]. None of the *Xylaria* species described have to date been studied regarding their biological properties. Therefore, in the current study ten isolates of novel *Xylaria* species were selected for extraction of bioactive compounds from the fermented broths. These were investigated for their antioxidant activities and cytotoxicity against three different cancer cell lines (human lung carcinoma (A549), liver (HepG2) and cervical (Hela)) compared with normal cells for evaluating their potential uses as medicinal fungi in future. The metabolic profiles of all extracts were also analyzed using LC-MS method.

## 2. Materials and Methods

### 2.1. Chemicals and Reagents

Butylated hydroxytoluene (BHT), RPMI 1640 stable glutamine medium, gallic acid, 2,2′-azino-bis(3-ethylbenzothiazoline-6-sulfonic acid) diammonium salt (ABTS), 2,2-diphenyl-1-picrylhydrazyl (DPPH), 6-hydroxy-2,5,7,8 tetramethylchroman-2-carboxylic acid (Trolox^®^), Folin–Ciocalteu’s reagent (Sigma-Aldrich, Burlington, MA, USA), acetic acid, D-glucose anhydrous, dimethyl sulfoxide (DMSO), ethyl acetate (analytical grade) (EtOAc), methanol (analytical grade) (MeOH), methanol (MS grade), water (MS grade) (Fisher Scientific, UK), malt extract, peptone, potato dextrose agar (PDA), yeast extract (Himedia, Mumbai, India), and sodium carbonate (Na_2_CO_3_) (Ajax, New Zealand) were used in the present study.

### 2.2. Fungal Materials

**Figure 1:**
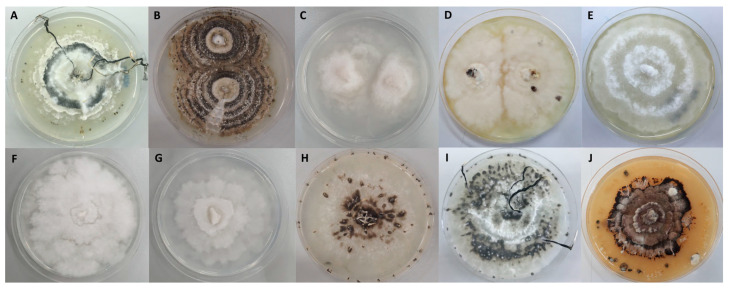
Cultures of *Xylaria* species associated with termite nests on PDA for extraction. (**A**) *X. subintraflava* SWUF16-11.1. (**B**) *X. subintraflava* SWUF17-24.2. (**C**) *X. siamensis* SWUF17-20.2. (**D**) *X. chaiyaphumensis* SWUF17-49.2. (**E**) *X. chaiyaphumensis* SWUF16-4.1. (**F**) *X. chaiyaphumensis* SWUF16-11.4. (**G**) *X. thienhirunae* SWUF16-44.1. (**H**) *X. thienhirunae* SWUF16-10.1. (**I**) *X. thienhirunae* SWUF16-7.2. (**J**) *X. vinacea* SWUF18-2.3.

The 10 *Xylaria* samples used in this study were obtained from the Department of Microbiology, Faculty of Science, Srinakharinwirot University, Bangkok, Thailand. They had been recently recorded as new species associated with termite nests and had been described as the following five species; *X. subintraflava* (SWUF16-11.1 and SWUF17-24.2), *X. siamemsis* (SWUF17-20.2), *X. chaiyaphumensis* (SWUF16-4.1, SWUF16-11.4, SWUF17-49.2), *X. thienhirunae* (SWUF16-7.2, SWUF16-10.1, SWUF17-44.1) and *X. vinacea* (SWUF18-2.3) (Figure 1). The cultures were isolated from the holotype and confirmed by nucleotide sequences, which were submitted in the GenBank database as described in Wangsawat et al. [19].

### 2.3. Fungal Cultivation and Extraction

The fungal isolates were cultured on PDA in 9 cm Petri dishes at 30 °C for 2 weeks. Mycelium on a PDA plate was cut into small pieces before being transferred into 200 mL of YM medium, and incubated at 30 °C for 6 weeks. Each cultural broth was filtrated and extracted with an equal volume of ethyl acetate three times. Then, the solvent was removed by evaporation under reduced pressure at 45 °C using a rotary evaporator. The crude extracts were stored in tightly caped glass bottles at −20 °C until used.

### 2.4. DPPH Scavenging Activity

The DPPH scavenging assay was modified from Xiang et al. [24]. Briefly, 55 microliters of each extract at different concentrations prepared by a two-fold dilution were added into 96–well plates. Then, 150 µL of 0.2 mM DPPH solution was mixed and incubated for 30 min in the dark. The absorbance was measured at 517 nm. Butylated hydroxytoluene (BHT) and Trolox were used as positive controls. All reactions were performed in triplicate (*n* = 3). The percentage of DPPH scavenging activity was calculated using the following equation:

DPPH radical scavenging (%) = [(Abs_control_ − Abs_sample_)/Abs_control_] × 100

where Abs_control_ is the absorbance of the control reaction and Abs_sample_ is the absorbance of the extract. IC_50_ value is the concentration of the extract required to inhibit 50% of DPPH radical scavenging. A scatter graph was plotted to obtain IC_50_ value.

### 2.5. ABTS Scavenging Activity

The ABTS scavenging activity was evaluated according to the method of Almeida et al. [25] with some modifications. A stock of ABTS radical cation (ABTS^•+^) was prepared by the reaction of 7 mM ABTS solution (5 µL) with 140 mM potassium persulfate (88 µL), and incubated for 16 h in the dark. The ABTS^•+^ solution was diluted with 95% ethanol to obtain absorbance 1.0 at 734 nm. Ten microliters of different concentrations of the extracts was added into 96–well plate, mixed with the ABTS^•+^ solution (290 µL), and incubated in the dark. After 7 min, the absorbance was measured at 734 nm. BHT and Trolox were used as positive controls. All samples were evaluated in triplicate. The % ABTS radical scavenging activity was calculated using the following equation:

ABTS radical scavenging (%) = [(Abs_control_ − Ab_ssample_)/Abs_control_] × 100

where Abs_control_ is the absorbance of the control reaction and Abs_sample_ is the absorbance of the extract. The antiradical activity was expressed as IC_50_ (mg/mL), which represented the extract concentrations scavenging 50% of ABTS radicals. IC_50_ values were calculated using Prism 8.0 calculated from the plotted graph between % scavenging and concentration.

### 2.6. Total Phenolic Content

Total phenolic content (TPC) was quantified using Folin–Ciocalteu method according to Rusu et al. [26] with some modifications. The stock of Folin–Ciocalteu’s reagent was prepared by Folin–Ciocalteu’s reagent with water (1:10, *v*/*v*). The extract (25 µL) was added into 96–well plate and mixed with 75 µL of 7.5% (*w*/*v*) of sodium carbonate (Na_2_CO_3_). After 3 min, 100 µL of the stock of Folin–Ciocalteu’s reagent was added and incubated for 30 min in the dark. The absorbance was measured at 765 nm. The TPC was expressed as g gallic acid equivalent per g of crude extract (g GAE/g). The experiments were evaluated in triplicate.

### 2.7. Cytotoxicity against Cancer Cell Lines

#### 2.7.1. Cell Culture

The cell lines of human lung carcinoma (A549), human liver carcinoma (HepG2) and human cervical adenocarcinoma (HeLa) were purchased from the American Type Culture Collection (ATCC), (LGC Standards, Middlesex, UK), and the immortalized human normal prostate cell line (PNT2) was obtained from the European Collection of Authenticated cell cultures (ECACC), (Public Health England, Salisbury, UK). All cells were cultured in 75 cm^2^ cell culture flasks in complete medium (RPMI 1640 stable Glutamine Medium supplemented with 10% fetal bovine serum (FBS) and 1% Pen/strep) at 37 °C in an atmosphere of 95% humidity and 5% CO_2_. After reaching 70–80% confluence, the cells were washed with phosphate buffer saline (PBS) and the cells dislodged using trypsin (1× trypsin-EDTA solution in PBS). The cells were then resuspended with fresh complete medium and used to seed new cell culture flasks. For experimental purposes, the cells were seeded (100 µL/well) into 96–well plates at differing concentrations (5 × 10^3^ cells/well for A549, HepG2 and HeLa cells, and 10 × 10^4^ cells/well for PNT2 cells).

#### 2.7.2. Assessment of the Cytotoxicity of *Xylaria* Species Crude Extracts against Cancer Cell Lines

The assessment of the cytotoxicity of the crude extracts was performed on cells growing in 96-well plates (as described above) using MTT (3-[4,5-dimethylthiazol-2-yl]-2,5-diphenyl-tetrazolium bromide) assay following Guetchueng et al. [27]. After seeding, the cells were first cultured overnight before treatment with the crude extracts. The cells were then treated with crude extracts (100 µg/mL (100 µL/well)) in complete medium with 0.2% (*v*/*v*) DMSO. In addition, cells were treated with complete medium (100 µL/well) as a negative control and doxorubicin (50 µg/mL (100 µL/well)) as a positive control. Both these controls also contained DMSO (0.2% (*v*/*v*)). After 48 hours’ treatment, cell viability was determined using the (MTT) assay. The treatments were removed from the wells and MTT in complete medium (0.5 mg/mL (100 µL/well)) was added and the cells incubated for further 2 h at 37 °C. This mixture was removed and 200 µL/well DMSO added to solubilize the formazan crystals. The plate was shaken slowly for 20 min then the absorbance of each well was measured at 570 nm. All crude extracts (and controls) were assessed in triplicate. The effect of each crude extract on the proliferation of cancer cells was expressed as percentage of the absorbance of the average negative control absorbance reading, which were calculated using the equation:

Cell viability (%) = (Abs_sample/_Abs_control_) × 100

where Abs_control_ is the absorbance of the control reaction and Abs_sample_ is the absorbance of the extract.

### 2.8. Statistical Analysis

All the assays were carried out in triplicate on each occasion. The data were presented as the mean ± standard deviation (SD). Differences between the individual treatments were analyzed using one-way ANOVA with Tukey’s method performed in SPSS programme version 26. Statistical significance was set at *p*-value < 0.05. IC_50_ values were calculated by using Graph Pad Prism (Version 8.0)

### 2.9. LC-MS Chemical Profiles

The chemical constituents of the extracts were determined using liquid chromatography coupled with mass spectrometry (LC-MS) analysis according to the method previously described by Khan et al. [28] with modifications. The Alliance HPLC system 2695 (water) was used. Chromatography was performed using reversed-phase on a Phenomenex C18 column (150 mm × 4.6 mm × 5 µm). The conditions were set as follows; column temperature at 25 °C, UV-Vis detector at 200–400 nm, 1 mL/min of flow rate and 20 µL of injection volume. An elution gradient was used 0.1% acetic acid in water as mobile phase A and 0.1% acetic acid in MeOH as mobile phase B. The mobile phase composition was started from 30% B at 0 min and increased as linear gradient reaching 100% B at 30 min. The LC system was connected to a quadrupole time of flight (TOF) mass spectrometer (Water Micromass LCT) having an electrospray ion source. The response was recorded in real time by the mass spectrometer data system (Waters MassLynx version 4.1). The parameters were set as follows: electrospray interface 3000 V, sample cone 60 V, extraction cone 4 V, rangefinder lens 300 V, desolvation temperature 250 °C, source temperature 100 °C, nebulizer gas flow 20 L/h, desolvation gas flow 760 L/h, and TOF tube 4687 V. The Data acquisition method was set as follows: cycle time 1 s, scan duration 0.9 s, inter-scan delay 0.1 s, mass range 100–1500, and centroid mode. Positive ion mode was operated.

## 3. Results

### 3.1. Determination of Antioxidant Activities

#### 3.1.1. DPPH Free Radical Scavenging Activity

**Figure 2:**
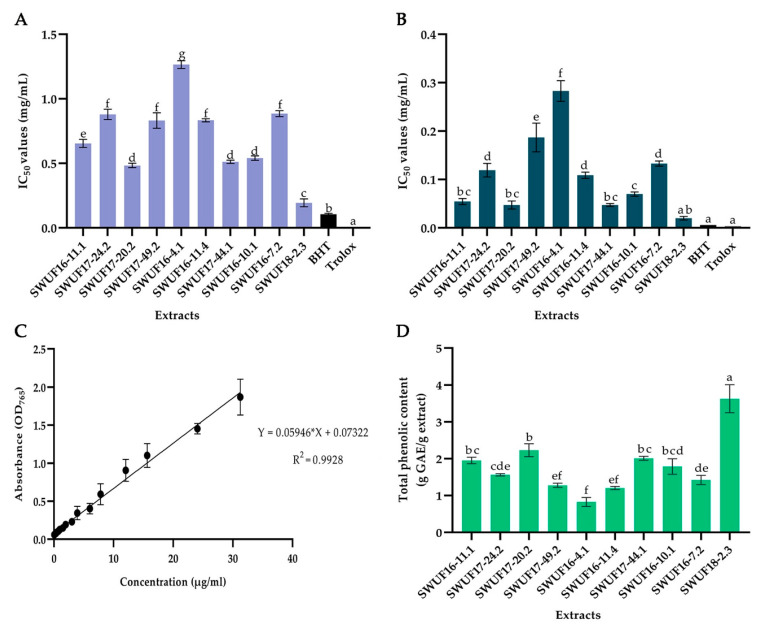
IC_50_ values of antioxidant activities, DPPH and ABTS radical scavenging and total phenolic content of ethyl acetate extracts of *Xylaria* spp. and standard BHT and Trolox. (**A**) DPPH assay. (**B**) ABTS assay. (**C**) standard curve of gallic acid. (**D**) total phenolic content. Data expressed as means ± standard deviation. Means within each graph with different letters (a–g) differ significantly (*p* < 0.05).

The DPPH free radical is a stable free radical, which has been widely used as a tool for estimating the free radical scavenging activities of antioxidants [29]. The DPPH scavenging activities of 10 *Xylaria* extracts with EtOAc are presented in Figure 2A (Table S1). All extracts represented moderate to high antioxidant activities demonstrated in term of IC_50_ values ranged from 0.194 ± 0.031 to > 1.00 mg/mL. The extracts from different isolates were also varied within the same species. The highest significant activity belonged to *X. vinacea* SWUF18-2.3 (0.194 ± 0.031 mg/mL), while most extracts including *X. siamensis* SWUF17-20.2, *X. thienhirunae* SWUF17-44.1 and SWUF16-10.1 showed moderate activities ranging from 0.484 ± 0.017 to 0.541 ± 0.018 mg/mL (Table S1). In addition, nearly all extracts had antioxidant activity with IC_50_ values less than 1 mg/mL except for *X. chaiyaphumensis* SWUF16-4.1 (>1.00 mg/mL), although they proved less effective than the standard BHT (0.105 ± 0.006) and Trolox (0.004 ± 0.000) examined.

#### 3.1.2. ABTS Free Radical Scavenging Activity

The radical scavenging activities of 10 extracts determined by ABTS scavenging assays are presented in Figure 2B (Table S1). The IC_50_ of all extracts obtained was in a range of 0.020 ± 0.004 to 0.283 ± 0.021 mg/mL. Similar to DPPH assay, *X. vinacea* SWUF18-2.3 has the highest activity (IC_50_ value 0.020 ± 0.004 mg/mL) from the other extracts, which were not different from the standard BHT (0.006 ± 0.000 mg/mL) and Trolox (0.003 ± 0.000 mg/mL). These indicated that SWUF18-2.3 had significantly high efficacy similar to the standards. Most extracts showed moderate antioxidant activity which ranged from 0.047 ± 0.003 to 0.119 ± 0.014 mg/mL, while the isolate of SWUF16-4.1 showed the lowest activity at 0.283 ± 0.021 mg/mL. However, the antioxidant efficacy of almost all the extracts showed a similar trend to the DPPH scavenging assay.

### 3.2. Determination of Total Phenolic Content

The content of total phenolic compounds of all extracts is presented in Figure 2D (Table S1). The results varied from 0.826 ± 0.123 to 3.629 ± 0.381 g GAE/g crude extract according to the equation using gallic acid as the standard curve (Y = 0.05946 * X + 0.07322 and R^2^ = 0.9928) (Figure 2C). The highest total phenolic content of all *Xylaria* extracts belonged to *X. vinacea* SWUF18-2.3 (3.629 ± 0.381 g GAE/g extract) followed by *X. siamensis* SWUF17-20.2 (2.232 ± 0.176 g GAE/g extract). The results showed correlation between antioxidant activities (DPPH and ABTS scavenging activities) and total phenolic contents. The high total phenolic content contributed to the high antioxidant activities. *Xylaria vinacea* SWUF18-2.3 was the highest both total phenolic content and antioxidant activities, while *X. chaiyaphumensis* SWUF16-4.1 was the lowest both total phenolic content and antioxidant activities.

### 3.3. Cytotoxicity against Cancer Cell Lines

**Figure 3:**
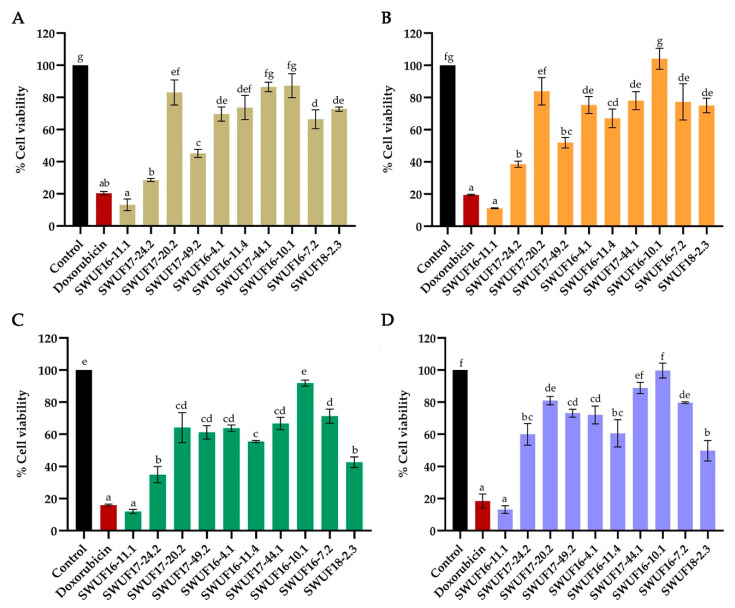
Cytotoxicity of EtOAc extracts of *Xylaria* spp. against four different cell lines at concentration of 100 μg/mL. (**A**) A549. (**B**) HepG2. (**C**) HeLa. (**D**) PNT2. Data expressed as means ± standard deviation. Means within each graph with different letters (a–g) differ significantly (*p* < 0.05).

Cytotoxicity of *Xylaria* extracts against different human cancer cell lines including A549, HepG2, HeLa and normal cell line PNT2—was determined using the MTT assay. The extracts showed varying cytotoxicity exhibited in the different cell lines at a concentration of 100 µg/mL (Figure 3 and Table S2). The ranges of % cell viability was 13.16 ± 3.59 – 87.26 ± 7.45% for A549, 11.15 ± 0.32 – 104.12 ± 6.55% for HepG2, 11.97 ± 1.28 – 91.82 ± 1.91% for HeLa and 13.17 ± 2.37 – 99.68 ± 4.67% for PNT2. Doxorubicin was used as the positive control having % cell viability of all cell lines ranging 15.99 ± 0.55 to 20.32 ± 1.09% at concentration of 50 µg/mL. The results revealed that most extracts exhibited cytotoxicity against at least one kind of the cancer cell lines. *Xylaria subintraflava* SWUF16-11.1 displayed the most significant inhibition to all cell lines with % cell viability ranged from 11.15 ± 0.32 to 13.17 ± 2.37. Some extracts revealed broad spectrum to inhibit several cancer cell lines, but some extracts were specific to only one cell lines. The extracts of *X. subintraflava* SWUF16-11.1 and SWUF17-24.2 showed highly significant activity against A549, HepG2 and HeLa while the extract of *X. vinacea* SWUF18-2.3 showed high cytotoxic activity against only HeLa cell (42.65 ± 3.32% cell viability). The extracts of *X. subintraflava* SWUF17-24.2 and *X. chaiyaphumensis* SWUF17-49.2 showed high cytotoxicity against cancer cell lines but low cytotoxicity against normal cell lines of PNT2. *Xylaria subintraflava* SWUF17-24.2 extract exhibited cytotoxicity against A549, HepG2 and HeLa ranged from 28.58 ± 0.95 to 38.54 ± 1.90% cell viability, while low cytotoxicity against PNT2 at 59.94 ± 6.76% cell viability. *Xylaria chaiyaphumensis* SWUF17-49.2 extract exhibited cytotoxicity against A549 cell lines at 45.14 ± 2.53 and 48.89 ± 7.51% cell viability, respectively, but showed low cytotoxicity against PNT2 at 73.10 ± 2.45 and 70.37 ± 12.46% cell viability, respectively.

### 3.4. LC-MS Chemical Profiles

**Figure 4:**
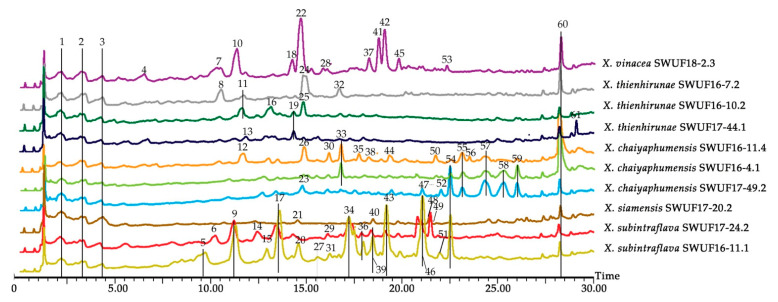
Total ion chromatograms (TIC) of EtOAc extracts from *Xylaria* culture broth.

LC-MS is one of the most effective analytical tools for organic compound analysis. The EtOAc extracts of secondary metabolites from *Xylaria* spp. were analysed to obtain LC chromatograms and MS spectrum using LC-ESI-QTOF MS method in positive mode. The gradient and condition of LC-MS analysis were optimized to separate and derive the highest peak numbers and the highest capacity of spectrum of the extracts. The results of total ion chromatograms (TIC) displayed more over 61 peaks of all extracts as shown in Figure 4 (Table S3). There were 18 tentatively identified compounds and 43 unidentified compounds when compared with mass bank database and previous literatures. The 18 identified compounds belonged to 14 groups of antimycin (no. 47), benzodiazepines (no. 28), diterpenoid (no. 22), diterpene (no. 13), cytochalasin (no. 9, 15, 17), cyclodepsipeptide (no. 59), flavones (no. 49, 50), flavonoids (no. 34), polyphenol (no. 29, 31), progesterone (no. 10), pyrone (no. 2), steroid derivatives (no. 1), triterpenoids (no. 23) and tropones (no. 19). The details of *m*/*z* spectra of each peak no. are described in Table S3. In this study, four major peaks of no. 1, 2, 3 and 60 were found in all *Xylaria* extracts, which presented at the same *m*/*z* spectrum and retention time. Peak no. 1 had a major ion at *m*/*z* 227.1 and minor peaks at *m*/*z* 197.1 and 261.1, which matched with prednosone in mass bank library. Peak no. 2 was predicted as xylaropyrone because of the highest [M + H]^+^ ion peak at *m*/*z* 211.1, which was similar to previous description by Siriwach et al. [30]. For peaks no. 3 and 60, their major peaks did not match any compounds in database including the previous reports. They were then noted as unidentified compounds, which might be new or mixed compounds representing in one peak.

The extracts of *X. subintraflava* SWUF16-11.1 and SWUF17-24.2 had the most numbers of peaks up to 19 and 16 peaks, respectively. The pattern of TIC and *m*/*z* spectrum were similar within the same species including both spectra of main peaks and retention times. Both extracts of *X. subintraflava* shared 12 major peaks (no. 1, 2, 3, 9, 17, 34, 36, 39, 43, 46, 47, 60) within the same species (Figure 4 and Table 1). Two peaks of no. 9 and 17 were predicted as 19,20-Epoxycytochalasin C or derivative, while peak no. 34 and 47 were predicted as naringin and animicin A, respectively (Table 1 and Table S3). For *X. chaiyaphumensis* extracts, three different extracts of SWUF16-4.1, SWUF16-11.4 and SWUF17-49.2 showed 6 main peaks (no. 1, 2, 3, 55, 57, 60) shared within species. Peak no. 55 and 57 did not matched any known compounds, then they were noted as unidentified compounds (Table S3). Interestingly, the extracts of *X. thienhirunae* SWUF16-7.2, SWUF16-10.1 and SWUF17-44.1 shared only 4 main peaks as other species. They showed different spectra in each isolate as given in Table 1. For other extracts of *X. siamensis* SWUF17-20.2 and *X. vinacea* SWUF18-2.3, that contained only one isolate, they had different main peak numbers. Among these extracts, *X. vinacea* SWUF18-2.3 had many main peaks up to 15 peaks (no. 1, 2, 3, 4, 7, 10, 18, 22, 28, 37, 41, 42, 45, 53 and 60) including shared 4 main peaks with other species. Peak no. 10 was predicted as 11-alpha-acetoxyprogesterone and peak no. 22 was andrographolide (Table 1 and Table S3). Although most of main peaks from *Xylaria* extracts in this study were designated as unidentified compounds, they are new reports of TIC pattern and *m*/*z* spectra needed to purify and study in further.

## 4. Discussion

The discovery of novel secondary metabolites from insect-associated fungi has been of interest to researchers in recent years [23,31,32,33]. Lately, twelve new species of *Xylaria* associated with termite nests or growing on soil were described by our previous study [19]. Ten isolates of 5 species, *X. chaiyaphumensis*, *X. siamensis*, *X. subintraflava*, *X. thienhirunae* and *X. vinacea*, were selected to assess their biological activities and secondary metabolite analysis. The antioxidant capacity of all EtOAc extracts from *Xylaria* species was determined by using the DPPH and ABTS radical scavenging assays. The results showed that all extracts had antioxidant potentials, especially *X. vinacea* SWUF18-2.3, which had the lowest IC_50_ values (0.020 ± 0.004 to 0.194 ± 0.031 mg/mL) and they were not significantly different from the standard BHT (0.006 ± 0.000 mg/mL) and Trolox (0.003 ± 0.000 mg/mL) by ABTS scavenging assay. The IC_50_ values of DPPH scavenging assay of all extracts were slightly higher than ABTS assay. It is because the DPPH method has more limitations and is characterised by a lower sensitivity [34]. The antioxidant activity results in this study are better than those reported for *X. nigripes*, which is a traditional medicinal fungus and is a *Xylaria* species associated with termite nests [35]. The extracts of *X. nigripes* obtained from various solid-state fermentations with ethanol solvent had IC_50_ values of DPPH radical scavenging activity ranged from 2.0 ± 0.1 to 2.7 ± 0.1 mg/mL. In addition, Ko et al. [21] reported that the DPPH activity of *X. nigripes* extracts from culture mycelium using different extraction solvents of aqueous and ethanol had IC_50_ 62.07 µg/mL and 73.49 µg/mL, respectively, while the commercial mycelium from a drug store in China known as “Wu Ling Shen” were extracted with the same solvents and showed the IC_50_ 487.68 µg/mL and >500 µg/mL, respectively. The extracts of all *Xylaria* species in this study showed strong antioxidant activities compared with other reports of *Xylaria* associated from termite nests, and they were noted as good sources of antioxidant activities for further study. However, the antioxidant capacities of fungal extracts are strongly dependent on the culture media and the extracting solvent, due to the presence of different antioxidant compounds of varied chemical characteristics and polarities that may or may not be soluble in a particular solvent.

The phenolic compounds present in several fungi and relate to biological functions including antioxidant activity. The total phenolic compounds of the *Xylaria* extracts in this study were high levels and varied from 0.826 ± 0.123 to 3.629 ± 0.381 g GAE/g crude extract. The high total phenolic contents and the strong antioxidant activities were from the same species *X. vinacea* SWUF18-2.3. It might be that of the different phenolic compounds contributed to the antioxidant activity. In a previous study, it has been reported that antioxidant activity from *Xylaria* spp. could be affected mainly by the presence of phenolic compounds. As the total phenolic compounds increase, the antioxidant potential of the fungi increases. This study was supported by previous studies which found that the levels of phenolic compounds resulted in their antioxidant capacity or that they correlated [35,36,37].

Fungal secondary metabolites have recently attracted great interest, owing to their versatile applications including cytotoxicity against cancer cells [38,39]. In our previous study, several active compounds extracted from *Xylaria* culture broth with EtOAc showed high cytotoxicity against different cancer cell lines [40]. McCloskey et al. [40] demonstrated that the cytotoxicity of chaxine C derived from EtOAc extract of *X. allantoidea* SWUF76 had strong effects against HeLa, HT29 colon cancer cell, HCT116 colon cancer cell, MCF-7 breast cancer cell and normal Vero cells. Moreover, two new tetralone derivatives named xylariol A and B derived from *X. hypoxylon* AT-028 exhibited cytotoxicity against HepG2 cell lines with IC_50_ of 22.3 and 21.2 μg/mL, respectively [41]. In this study, the cytotoxicity of *Xylaria* extracts showed different potential to cancer cell lines, although within the same species. The extracts of *X. subintraflava* SWUF16-11.1 exhibited the strongest cytotoxicity against all cancer cell lines at the concentration of 100 μg/mL, but SWUF17-24.2 showed less activity than those isolates within the same species. For *X*. *chaiyaphumensis* isolates, only SWUF17-49.2 showed % cell viability against A549 less than 50%, while other isolates of SWUF16-4.1 and SWUF16-11.4 showed higher % cell viability. These results could be because of difference in responsiveness and sensitivity of different cancer cells to different compounds [42]. Liu et al. [43] reported that different phenanthroindolizidine alkaloids (PAs) revealed difference of one ethoxyethyl on molecule resulting in different results of inhibition which showed greater effects on HepG2 compared with HCT116 and HT29 cancer cells. The extracts of this study were crude extracts containing several compounds, which may have been the results of synergistic or antagonistic effects to different cancer cell lines. In addition, the extracts of some isolates revealed relationships between cytotoxicity and antioxidant activities. The high cytotoxicity of *X. subintraflava* SWUF16-11.1 extract had strong antioxidant activities and high content of total phenolic compounds. In contrast, the extract of *X. vinacea* SWUF18-2.3 showed low toxicity but it contained high antioxidant activity. Therefore, the increasing of antioxidant activities of some extracts did not affect to the cytotoxicity against cancer cells. However, until now, only one study has proved that the extracts of *Xylaria* species from termite nests have anticancer activity [31]. Regarding their interesting biological activities and presence of highly cytotoxic metabolites, which may be a potential source of leads for drug development after more extensive pharmacological screening of the isolated compounds e.g., target identification and validation, preclinical studies and clinical development.

Currently, metabolomics have been utilized in medicinal and pharmaceutical technology for the analysis of their quality and components. An untargeted metabolomics analysis simultaneously detects as many metabolites as possible, to systematically compare the features of metabolites among species. The LC-MS approach is a powerful tool for studying metabolites, including distinguishing the compound compositions and characterising chemical structures [44,45,46]. The derived fingerprint chromatograms from this study demonstrated the possible secondary metabolite compositions from *Xylaria* crude extracts. Only four species of *Xylaria* from termite nests, *X. acuminatilongissima* YMJ623, *X. fimbriata* YMJ491, *X. nigripes* and *Xylaria* sp., have been observed for their metabolic compounds including biological activities [23,31,32,33]. The main chemical constituents of their metabolites were aromatic compounds (benzofurans, benzohydrofuran, carbinol, coumarins, isoprenyl phenolic ethers, naphthalene glycoside, phthalides and tetralone derivatives), alkaloids (spirocyclic pyrrole alkaloids), cyclopeptides (carboxylic acids and derivatives), polyketides (including acremines) and terpenoids (sesquiterpene acids, sesquiterpenoids) [23,31,32,33,47,48,49,50,51]. In the present study, 18 identified compounds belong to a variety of groups of compounds and most of the compounds are first recorded for the first time from *Xylaria* species on termite nests including aromatic compounds or alkaloid (animicin A, colchicine), cyclopeptide (bassianolide), flavonoids (naringin, tiliroside, 4′-O-(2′-Z-Feruloyl GluA(1-2)Glu A) apigenin), N-containing compounds (tofisopam), polyphenol compound (rottlerin), steroids (prednisone, 11-alpha-acetoxyprogesterone) and terpenoids (andrographolide, euphyperin B, lagochilin).

The results of TIC profiles in our study shared four main peaks (no. 1, 2, 3 and 60) within all *Xylaria* extracts. One main peak, no. 2 was identified as xylaropyrone, which was previously reported from endophytic *X. feejeensis* [30] and xylaropyrone derivatives (B and C) from endophytic *Xylaria* sp. SC1440 [52]. These compounds showed moderate antimicrobial activity against *Saccharomyces cerevisiae*, *Escherichia coli*, *Pseudomonas aeruginosa* and *Staphylococcus aureus* at different concentrations [30]. For the other 3 main peaks, peak no. 1 matched to prednisone, which is a group of steroids having anti-inflammatory and immunomodulation properties, while other 2 main peaks no. 3 and 60 were unidentified compounds that may be new or complex compounds and need to be purified to determine their chemical structure by analysis in a further study. In addition, each *Xylaria* isolate provided differences in their metabolic characteristics or spectrum profiles. This may be because of their genetic variations combined with the different locations and soil properties of each isolate. The previous studies by Tang et al. [53] and Li et al. [54] reported that the metabolic compounds obtained from microorganism were complex and the metabolic accumulation was variable due to the great influences of genetic and environmental factors. Most spectra of mass patterns obtained from *Xylaria* EtOAc extracts in the present study exhibited the different peaks of mass at the same retention times, which implied that those peaks cannot be representative in discriminating the different species. Due to the complex of ion spectrum and the limitation of fungal metabolites in mass databases, many peaks were noted as unidentified compounds. In addition, all extracts obtained from novel *Xylaria* species associated with termite nests (subgenus *Pseudoxylaria*), which have revealed a separate lineage from other *Xylaria* species associated with different habitats analyzed by phylogenetic analysis [18,19]. Therefore, the metabolites from such a termite-associated species have only received a few reports and proved to be rather unique cyclic tetrapeptides [55], which were different from the regular *Xylaria* species living as saprophytes or endophytes [56]. The highest number of peaks belonged to *X. subintraflava* SWUF16-11.1 followed by *X. vinacea* SWUF18-2.3, which exhibited the highest antioxidant activities and cytotoxicity against all cancer cell lines. The results may be related to the most peak numbers of the extracts from LC-MS fingerprints. The various groups of identified compounds found in the present study have been reported as processing biological activities including antimicrobial, anti-viral, antioxidant, anti-inflammatory, anticancer, anti-tumor and anti-insecticidal activities [10,13,22,56]. For example, bassianolide (peak no. 59) from *X. chaiyaphumensis* SWUF16-4.1 and SWUF17-49.2 was firstly reported from a wood-rot fungus *Xylaria* sp. BCC1067 and contained antiplasmodial, antimycobacterial and antitumor activities [57]. A 19,20-epoxy cytochalasin C (peak no. 9, 17) from *X. subintraflava* SWUF16-11.1 and SWUF17-24.2 has been previously found from *X. obovata* [58], endophyte *Diaporthe* sp. RJ-47 [59] and *Nemania* sp. UM10M, which has antimalarial activity and cytotoxicity against SK-MEL cell lines [60]. Some identified compounds andrographolide (peak no. 22) [61], naringin (peak no. 34) [62], rottlerin (peak no. 29) [63,64] tiliroside (peak no. 49) [65,66] have been reported from plants and contained anti-inflammatory, anti-cancer, anti-hepatotoxicity, anti-atherosclerosis, anti-diabetes and antioxidant activity. In addition, tofisopam (peak no. 28) from the extracts of *X. vinacea* SWUF18-2.3 was reported as synthetic compounds and has anxiolytic activity [67] and recently reported as post COVID neuro-psychiatric sequelae [68]. Isolation of new species of fungal metabolites may lead to the development of a new class of anticancer drugs through further study. Nevertheless, there were no report of LC-MS fingerprints derived from *Xylaria* spp. including *Xylaria* species associated from termite nests or soil. This is the first preliminary study of untargeted metabolic profiles which have contributed some important data about secondary metabolite compositions from the novel species of *Xylaria* from termite nests or soil.

## 5. Conclusions

Ethyl acetate extracts of *Xylaria* species associated with termite nests or soil possessed strong antioxidant activities and cytotoxicity against human cancer cell lines. The antioxidant ability may be from the action of phenolic compounds confirmed by total phenolic content experiment. The cytotoxicity exhibited a broad spectrum to specific activity against cancer cell lines. Moreover, LC-MS fingerprints presented over 61 peaks from ten isolates from six species which indicated that there were a many groups of secondary metabolites produced. The current study provided valuable information regarding the novel *Xylaria* species associated with termite nests or soil which have received limited attention concerning their biological activities and LC-MS fingerprints. Isolation and elucidation of bioactive chemical structures are now required for further study. However, the interesting activities may lead to those *Xylaria* associated with termite nests or soil becoming promising species of natural product sources for future therapeutic agents.

## Figures and Tables

**Table 1:** Peak numbers and identified compounds of *Xylaria* EtOAc extracts obtained from database comparison.

Species	Isolation ID.	Peak No.*	Identified Compound
*X. chaiyaphumensis*	SWUF16-4.1	**1**, **2**, 3, 33, 54, 55, 57, 58, **59**, 60	prednisone (1), xylaropyrone (2), bassianolide (59)
*X. chaiyaphumensis*	SWUF16-11.4	**1**, **2**, 3, 12, 26, 30, 33, 35, 38, 44, **50**, 55, 56, 57, 60	prednisone (1), xylaropyrone (2), 4′-O-(2′-Z-feruloyl GluA(1-2)GluA) apigenin (50)
*X. chaiyaphumensis*	SWUF17-49.2	**1**, **2**, 3, **23**, **47**, 52, 54, 55, 57, 58, **59**, 60	prednisone (1), xylaropyrone (2), euphyperin B (23), animicin A (47), bassianolide (59)
*X. subintraflava*	SWUF16-11.1	**1**, **2**, 3, 5, **9**, **15**, **17**, 20, 27, **31**, **34**, 36, 39, 43, 46, **47**, 51, 54, 60	prednisone (1), xylaropyrone (2), 19,20-epoxy cytochalasin C or derivative (9, 15, 17), rottlerin (31), naringin (34), animicin A (47)
*X. subintraflava*	SWUF17-24.2	**1**, **2**, 3, 6, **9**, 14, **17**, **29**, **34**, 36, 39, 43, 46, **47**, **49**, 60	prednisone (1), xylaropyrone (2), 19,20-epoxy cytochalasin C or derivative (9, 17), rottlerin (29), naringin (34), animicin A (47), tiliroside (49)
*X. thienhirunae*	SWUF16-7.2	**1**, **2**, 3, 8, 11, 24, 32, 60	prednisone (1), xylaropyrone (2)
*X. thienhirunae*	SWUF16-10.1	**1**, **2**, 3, 11, 16, **19**, 25, 60	prednisone (1), xylaropyrone (2), colchicine (19)
*X. thienhirunae*	SWUF17-44.1	**1**, **2**, 3, **13**, **19**, 60, 61	prednisone (1), xylaropyrone (2), lagochilin (13), colchicine (19)
*X. siamensis*	SWUF17-20.2	**1**, **2**, 3, 21, 40, 48, 60	prednisone (1), xylaropyrone (2)
*X. vinacea*	SWUF18-2.3	**1**, **2**, 3, 4, 7, **10**, 18, **22**, **28**, 37, 41, 42, 45, 53, 60	prednisone (1), xylaropyrone (2), 11-alpha-acetoxyprogesterone (10), andrographolide (22), tofisopam (28)

* The peak numbers labelled in bold are identified compounds.

## Data Availability

Data is contained within the article.

## Supplementary Materials

The following are available online at https://www.mdpi.com/article/10.3390/antiox10101557/s1, Table S1: IC_50_ values of antioxidant activities, DPPH and ABTS radical scavenging and total phenolic content of ethyl acetate extracts of *Xylaria* spp. and standard BHT and Trolox. Data expressed as means ± standard deviation. Means within each graph with different letters (a–g) differ significantly (*p* < 0.05), Table S2: Cytotoxicity of EtOAc extracts of *Xylaria* spp. against four different cell lines, A549, HepG2, HeLa and PNT2, at concentration of 100 μg/mL. Data expressed as means ± standard deviation. Means within each graph with different letters (a–g) differ significantly (*p* < 0.05), Table S3: Mass spectra data of peaks from *Xylaria* species and identified compounds.
