# Supplementary material for: Antioxidant Activity and Cytotoxicity against Cancer Cell Lines of the Extracts from Novel Xylaria Species Associated with Termite Nests and LC-MS Analysis
Source: Antioxidants (Basel). 2021 Sep 29;10(10):1557. doi: 10.3390/antiox10101557 (PMC8533195; doi:10.3390/antiox10101557)
Supplement: Supplementary file 1 [file antioxidants-10-01557-s001.zip › antioxidants-1386172-supplementary.pdf]

Supplementary Materials

Table S1: IC_{50} values of antioxidant activities, DPPH and ABTS radical scavenging and total phenolic content of ethyl acetate extracts of *Xylaria* spp. and standard BHT and Trolox. Data expressed as means ± standard deviation. Means within each graph with different letters (a–g) differ significantly (P < 0.05)

Samples	DPPH IC_{50} value (mg/ml)	ABTS IC_{50} value (mg/ml)	TPC (g GAE/g extract)
X. subintraflava SWUF16-11.1	0.654 ± 0.032^{e}	0.054 ± 0.006^{bc}	1.954 ± 0.088^{bc}
X. subintraflava SWUF17-24.2	0.879 ± 0.040^{f}	0.119 ± 0.014^{d}	1.563 ± 0.031^{cde}
X. siamensis SWUF17-20.2	0.484 ± 0.017^{d}	0.047 ± 0.008^{bc}	2.232 ± 0.176^{b}
X. chaiyaphumensis SWUF17-49.2	0.831 ± 0.060^{f}	0.187 ± 0.030^{e}	1.278 ± 0.058^{ef}
X. chaiyaphumensis SWUF16-4.1	$>1.00^{g}$	0.283 ± 0.021^{f}	0.826 ± 0.123^{f}
X. chaiyaphumensis SWUF16-11.4	0.834 ± 0.012^{f}	0.109 ± 0.006^{d}	1.204 ± 0.043^{ef}
X. thienhirunae SWUF17-44.1	0.512 ± 0.012^{d}	0.047 ± 0.003^{bc}	2.009 ± 0.054^{bc}
X. thienhirunae SWUF16-10.1	0.541 ± 0.018^{d}	0.070 ± 0.004^{c}	1.789 ± 0.211^{bcd}
X. thienhirunae SWUF16-7.2	0.885 ± 0.022^{f}	0.133 ± 0.005^{d}	1.423 ± 0.124^{de}
X. vinacea SWUF18-2.3	0.194 ± 0.031^{c}	0.020 ± 0.004^{ab}	3.629 ± 0.381^{a}
BHT	0.105 ± 0.006^{b}	0.006 ± 0.000^{a}	-
Trolox	0.004 ± 0.000^{a}	0.003 ± 0.000^{a}	-

Data expressed as means ± standard deviation. Means within each column with different letters (a–g) differ significantly (P < 0.05).

Table S2: Cytotoxicity of EtOAc extracts of *Xylaria* spp. against four different cell lines, A549, HepG2, Hela and PNT2, at concentration of 100 μg/mL. Data expressed as means ± standard deviation. Means within each graph with different letters (a–g) differ significantly (P < 0.05)

**Samples**	**% Cell viability**			
	**A549**	**HepG2**	**HeLa**	**PNT2**
X. subintraflava SWUF16-11.1	13.16 ± 3.59^{a}	11.15 ± 0.32^{a}	11.97 ± 1.28^{a}	13.17 ± 2.37^{a}
X. subintraflava SWUF17-24.2	28.58 ± 0.95^{b}	38.54 ± 1.90^{b}	34.86 ± 5.05^{b}	59.94 ± 6.76^{bc}
X. siamensis SWUF17-20.2	83.09 ± 7.79^{ef}	83.85 ± 8.50^{ef}	64.15 ± 9.35^{cd}	80.95 ± 2.63^{de}
X. chaiyaphumensis SWUF17-49.2	45.14 ± 2.53^{c}	51.92 ± 3.27^{bc}	61.20 ± 4.20^{cd}	73.10 ± 2.45^{cd}
X. chaiyaphumensis SWUF16-4.1	69.64 ± 4.37^{de}	75.38 ± 5.30^{de}	63.74 ± 2.02^{cd}	72.00 ± 5.55^{cd}
X. chaiyaphumensis SWUF16-11.4	73.66 ± 7.56^{def}	67.02 ± 5.81^{cd}	55.46 ± 0.60^{c}	60.57 ± 8.52^{bc}
X. thienhirunae SWUF17-44.1	86.47 ± 2.97^{fg}	78.05 ± 5.58^{de}	66.69 ± 3.83^{cd}	88.84 ± 3.48^{ef}
X. thienhirunae SWUF16-10.1	87.26 ± 7.45^{fg}	104.12 ± 6.55^{g}	91.82 ± 1.91^{e}	99.68 ± 4.67^{f}
X. thienhirunae SWUF16-7.2	66.44 ± 5.85^{d}	77.28 ± 11.26^{de}	71.23 ± 4.41^{d}	79.67 ± 0.57^{de}
X. vinacea SWUF18-2.3	72.72 ± 1.33^{de}	75.06 ± 4.47^{de}	42.65 ± 3.32^{b}	49.78 ± 6.32^{b}
Doxorubicin	20.32 ± 1.09^{ab}	19.47 ± 0.37^{a}	15.99 ± 0.55^{a}	18.47 ± 4.40^{a}
Medium	100.00^{g}	100.00^{fg}	100.00^{e}	100.00^{f}

Data expressed as means ± standard deviation. Means within each column with different letters (a–g) differ significantly (P < 0.05).

Table S3: Mass spectra data of peaks from *Xylaria* species and identified compounds

Peak No.	t_R (min)	MS data (m/z)	Isolates	m/z of predicted compounds	Predicted compounds	Group of compounds	References
**1**	2.14-2.20	197.1, **227.1**, 261.1	all		prednisone	steroids derivatives	
**2**	3.19-3.42	183.1, **211.1**	all		xylaropyrone	pyrone	
**3**	4.24-4.35	154.0, 217.1, **245.1**, 331.0	all		unknown 1		
**4**	6.54	**233.0**, 389.1, 411.0, 799.1	SWUF18-2.3		unknown 2		
**5**	9.31-9.72	402.2, **556.2**, 562.2, 578.2, 1103.4, 1133.4	SWUF16-11.1		unknown 3		
**6**	10.27	217.1, **245.1**, 313.1	SWUF17-24.2		unknown 4		
**7**	10.32	185.0, **222.1**, 268.1, 302.0, 375.1	SWUF18-2.3		unknown 5		
**8**	10.52	151.0, 183.1, 225.1, 285.1, **331.1**	SWUF16-7.2		unknown 6		
**9**	11.22-11.29	404.2, **428.2**, 446.2, 524.2, **546,2**, 1069.5	SWUF16-11.1, SWUF17-24.2	524.2, $[M+H]^+$ 546.2, $[M+Na]^+$	19,20-epoxy cytochalasin C or derivative	cytochalasin	[1]
**10**	11.37	233.0, 373.1,	SWUF18-2.3		11-alpha-acetoxyprogesterone	progesterone	

Peak No.	t_R (min)	MS data (m/z)	Isolates	m/z of predicted compounds	Predicted compounds	Group of compounds	References
		**395.0**, 767.1					
**11**	11.67-11.69	173.0, **285.1**, **343.1**, 469.1, 627.1	SWUF16-10.1, SWUF16-7.2		unknown 7		
**12**	11.71	173.0, **309.1**, 469.1, 627.1	SWUF16-11.4		unknown 8		
**13**	11.84	267.1, 285.1, **357.2**	SWUF17-44.1		lagochilin	diterpene	
**14**	12.46	**188.1**, **343.2**, 665.4, 695.3	SWUF17-24.2		unknown 9		
**15**	12.86-12.94	370.1, 404.2 524.2, **546.2**, 562.2, 1069.5	SWUF16-11.1	524.2, $[M+H]^+$ 546.2, $[M+Na]^+$	19,20-epoxy cytochalasin C or derivative	cytochalasin	[1]
**16**	13.18	223.1, 322.2, 325.1, **385.2**, 398.2	SWUF16-10.1		unknown 10		
**17**	13.48-13.61	404.2, 428.2 446.2, 524.2, **546.2**, 562.2, 1069.5	SWUF16-11.1, SWUF17-24.2	524.2, $[M+H]^+$ 546.2, $[M+Na]^+$	19,20-epoxy cytochalasin C or derivartive	cytochalasin	[1]
**18**	14.28	287.1, **373.1**	SWUF18-2.3		unknown 11		
**19**	14.34-14.38	169.1, 225.1, 285.1, **325.1**, 357.2	SWUF17-44.1, SWUF16-10.1		colchicine	tropones	

**Peak No.**	***tR* (min)**	**MS data (m/z)**	**Isolates**	**m/z of predicted compounds**	**Predicted compounds**	**Group of compounds**	**References**
**20**	14.51-14.58	418.2, 434.3, **597.3**, 619.3	SWUF16-11.1		unknown 12		
**21**	14.53	**263.1**, 327.1, 437.2	SWUF17-20.2		unknown 13		
**22**	14.71	287.1, 315.1, **701.2**, 1051.4, 1401.5	SWUF18-2.3		andrographolide	diterpenoid	
**23**	14.78	196.0, 352.2, **431.2**, 453.2, 883.4	SWUF17-49.2	431.2 $[M + H]^{+}$, 883.5 $[2 M + Na]^{+}$	euphyperin B	triterpenoids	[2]
**24**	14.81	**287.2, 345.0,** 437.2, 501.2, 675.2	SWUF16-7.2		unknown 14		
**25**	14.86	321.2, **357.2**, 437.2	SWUF16-10.1		unknown 15		
**26**	14.91	287.1, **509.2,** 675.2, 995.5	SWUF16-11.4		unknown 16		
**27**	15.54-15.59	265.1, 402.2, 430.2, **530.2,** 546.2, 1037.5	SWUF16-11.1		unknown 17		
**28**	15.89	**367.2**, **383.2**	SWUF18-2.3		tofisopam	benzodiazepines	
**29**	16.14	151.1, 243.1, 370.2, **517.3**, **539.3**	SWUF17-24.2		rottlerin	polyphenol	

Peak No.	t_R (min)	MS data (m/z)	Isolates	m/z of predicted compounds	Predicted compounds	Group of compounds	References
**30**	16.21	177.1, 269.1, 297.1, **315.1**, 369.2	SWUF16-11.4		unknown 18		
**31**	16.23	151.1, 243.1, 370.2, **517.3**, **539.3**	SWUF16-11.1		rottlerin	polyphenol	
**32**	16.69	269.1, **287.1**, 437.2	SWUF16-7.2		unknown 19		
**33**	16.76-16.84	271.2, **317.2**	SWUF16-4.1, SWUF16-11.4		unknown 20		
**34**	17.19-17.21	275.2, 402.2, **581.2**, **603.3**	SWUF16-11.1, SWUF17-24.2		naringin	flavonoids	
**35**	17.78	231.1, **475.2**, 927.5	SWUF16-11.4		unknown 21		
**36**	17.91-17.98	151.1, 284.3, **389.1**	SWUF16-11.1, SWUF17-24.2		unknown 22		
**37**	18.28	**369.2**, 747.2	SWUF18-2.3		unknown 23		
**38**	18.3	231.1, 284.3, **475.2**, 927.5	SWUF16-11.4		unknown 24		
**39**	18.41-18.48	243.1, 370.2, **585.3**, 607.3	SWUF16-11.1, SWUF17-24.2		unknown 25		
**40**	18.48	284.3, 385.2, **425.2**	SWUF17-20.2		unknown 26		

Peak No.	t_R (min)	MS data (m/z)	Isolates	m/z of predicted compounds	Predicted compounds	Group of compounds	References
**41**	18.78	**367.2,** 743.4	SWUF18-2.3		unknown 27		
**42**	19.08	**369.2**, 693.5, 747.4, 1039.7	SWUF18-2.3		unknown 28		
**43**	19.15-19.18	275.1, **565.3, 649.**3, 671.3	SWUF16-11.1, SWUF17-24.2		unknown 29		
**44**	19.36	**299.1, 371.2**	SWUF16-11.4		unknown 30		
**45**	19.83	**389.1, 489.1**	SWUF18-2.3		unknown 31		
**46**	19.83-19.90	181.1, **296.2**	SWUF 16-11.1, SWUF17-24.2		unknown 32		
**47**	20.83	402.3, **549.3, 571.3**	SWUF17-24.2, SWUF16-11.1, SWUF17-49.2		animicin A	antimycin	
**48**	21.43	**268.2, 591.3, 613.3**	SWUF17-20.2		unknown 33		
**49**	21.5	304.2, 309.2, **595.4**	SWUF17-24.2		tiliroside	flavones	
**50**	21.78	**797.4, 819.4**	SWUF16-11.4		4'-O-(2'-Z-Feruloyl GluA(1-2)GluA) apigenin (NMR)	flavones	
**51**	21.95	**425.2,** 443.2, 625.3, 1205.7	SWUF16-11.1		unknown 34		
**52**	22.01	**603.3, 625.3,** 694.4	SWUF17-49.2		unknown 35		

Peak No.	t_R (min)	MS data (m/z)	Isolates	m/z of predicted compounds	Predicted compounds	Group of compounds	References
**53**	22.35	233.0, **353.2**	SWUF18-2.3		unknown 36		
**54**	22.48-22.53	402.2, 470.3, **617.3, 639.3**, 1255.7	SWUF16-11.1, SWUF17-49.2, SWUF16-4.1		unknown 37		
**55**	23.11-23.16	**825.5, 847.5**	SWUF17-49.2, SWUF16-4.1, SWUF16-11.4		unknown 38		
**56**	23.55	231.1, **489.2**	SWUF16-11.4		unknown 39		
**57**	24.28-24.33	**853.5, 875.5**	SWUF17-49.2, SWUF16-4.1, SWUF16-11.4		unknown 40		
**58**	25.28-25.30	**881.5, 903.5**	SWUF17-49.2, SWUF16-4.1		unknown 41		
**59**	25.98	**355.2, 909.6,** 931.6	SWUF17-49.2, SWUF16-4.1	[M+Na]+ m/z 931.6	bassianolide	cyclodepsipeptide	[3]
**60**	28.20-28.32	181.0, 195.0, **413.2**, 445.3, 803.5	all		unknown 42		
**61**	29.13	**338.3**, 360.3	SWUF17-44.1		unknown 43		

References

1. Abate, D.; Abraham, W.-R.; Meyer, H. Cytochalasins and phytotoxins from the fungus *Xylaria obovata*. *Phytochemistry* **1997**, *44*, 1443–1448, doi:10.1016/S0031-9422(96)00780-7.
2. Zhao, J.X.; Shi, S.S.; Sheng, L.; Li, J.; Yue, J.M. Terpenoids and Steroids from *Euphorbia hypericifolia*. *Nat. Prod. Commun.* **2015**, *10*, 2049–2052.
3. Jirakkakul, J.; Punya, J.; Pongpattanakitshote, S.; Paungmoung, P.; Vorapreeda, N.; Tachaleat, A.; Klomnara, C.; Tanticharoen, M.; Cheevadhanarak, S. Identification of the nonribosomal peptide synthetase gene responsible for bassianolide synthesis in wood-decaying fungus *Xylaria* sp. BCC1067. *Microbiology* **2008**, *154*, 995–1006, doi:10.1099/mic.0.2007/013995-0.
